# Orientation of a bipolar membrane determines the dominant ion and carbonic species transport in membrane electrode assemblies for CO_2_ reduction†

**DOI:** 10.1039/d0ta12398f

**Published:** 2021-03-11

**Authors:** Marijn A. Blommaert, Rezvan Sharifian, Namrata U. Shah, Nathan T. Nesbitt, Wilson A. Smith, David A. Vermaas

**Affiliations:** Department of Chemical Engineering, Delft University of Technology 2629 HZ Delft The Netherlands d.a.vermaas@tudelft.nl; Wetsus, European Centre of Excellence for Sustainable Water Technology 8911 MA Leeuwarden The Netherlands

## Abstract

A bipolar membrane (BPM), consisting of a cation and an anion exchange layer (CEL and AEL), can be used in an electrochemical cell in two orientations: reverse bias and forward bias. A reverse bias is traditionally used to facilitate water dissociation and control the pH at either side. A forward bias has been proposed for several applications, but insight into the ion transport mechanism is lacking. At the same time, when implementing a BPM in a membrane electrode assembly (MEA) for CO_2_ reduction, the BPM orientation determines the environment of the CO_2_ reduction catalyst, the anolyte interaction and the direction of the electric field at the interface layer. In order to understand the transport mechanisms of ions and carbonic species within a bipolar membrane electrode assembly (BPMEA), these two orientations were compared by performing CO_2_ reduction. Here, we present a novel BPMEA using a Ag catalyst layer directly deposited on the membrane layer at the vapour–liquid interface. In the case of reverse bias, the main ion transport mechanism is water dissociation. CO_2_ can easily crossover through the CEL as neutral carbonic acid due to the low pH in the reverse bias. Once it enters the AEL, it will be transported to the anolyte as (bi)carbonate because of the presence of hydroxide ions. When the BPM is in the forward bias mode, with the AEL facing the cathode, no net water dissociation occurs. This not only leads to a 3 V lower cathodic potential but also reduces the flux of carbonic species through the BPM. As the pH in the AEL is higher, (bi)carbonate is transported towards the CEL, which then blocks the majority of those species. However, this forward bias mode showed a lower selectivity towards CO production and a higher salt concentration was observed at the cathode surface. The high overpotential and CO_2_ crossover in reverse bias can be mitigated *via* engineering BPMs, providing higher potential for future application than that of a BPM in forward bias owing to the intrinsic disadvantages of salt recombination and poor faradaic efficiency for CO_2_ reduction.

## Abstract 2

To study the transport mechanisms and effect of the orientation of a bipolar membrane, a novel bipolar membrane electrode assembly was created, indicating that reverse bias is most favourable for CO_2_ reduction.

## Graphical abstract

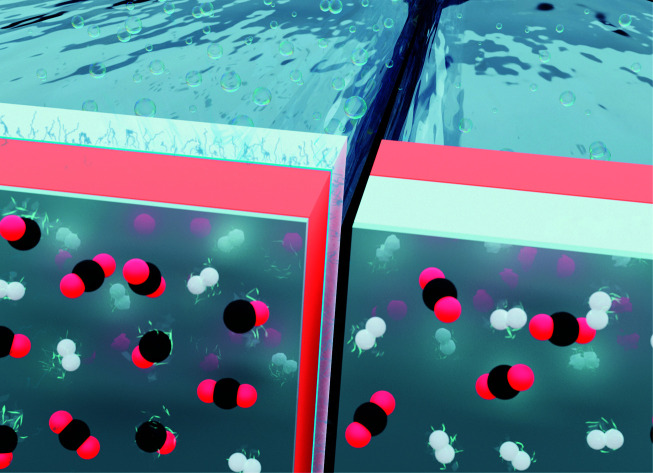

## Introduction

Electrochemical CO_2_ reduction using renewable energy sources is a key element in closing the carbon cycle while still providing carbon-based fuels and chemicals.^1^ The products from this reaction are chemical building blocks, which can be used in a wide variety of fuels and plastics. In order to be competitive with current industrial technologies, a high selectivity and throughput need to be achieved. In recent years, the technique of combining an electrode with a membrane, creating a membrane electrode assembly (MEA), has led to great improvements in the CO_2_ reduction field by achieving high selectivity and current densities relevant for industrial application.^2–5^ MEAs have intrinsic advantages to upscale CO_2_ reduction electrolysers, as they allow operation in a gas–liquid configuration (improving the CO_2_ concentration and mass transport towards the catalyst) while ensuring product separation. Different types of ion exchange membranes can be used in such MEA configurations, among which a cation exchange membrane (CEM)^6,7^ or an anion exchange membrane (AEM) are the most used in CO_2_ electrolyzers.^2–5^ A third type of membrane used in an MEA is a bipolar membrane (BPM), consisting of a cation and an anion exchange layer (CEL and AEL, respectively) with an internal interface between the two layers where a catalyst is deposited to enhance the possible water dissociation.^8–10^ In addition to the catalyst at the internal interface, electrolyte composition,^11^ and pH gradient,^12^ the two-layer configuration of the BPM allows us to choose the orientation of the membrane in an electrochemical cell.

**Fig. 1:**
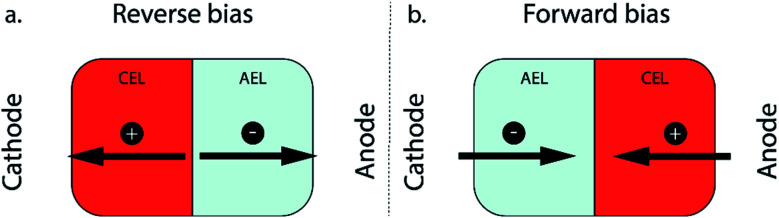
Modes of operation of a BPM, consisting of a cation exchange layer (CEL) and an anion exchange layer (AEL): (a) reverse bias and (b) forward bias.

For a monopolar membrane (*e.g.*, AEM or CEM), the orientation of the membrane has no impact on its function. For a BPM, the orientation of the membrane, determining which membrane layer faces the cathode, has great implications for its ion transport mechanism. Two modes of operation are possible with a BPM: reverse and forward bias (Fig. 1).

The first mode of operation is reverse bias with the CEL facing the cathode, where ions are depleted at the internal bipolar membrane interface upon applying a current. To fulfil the requirement of a current throughout the cell, ions need to be formed *via* the water dissociation reaction (WDR) into H^+^ and OH^−^ ions.^13^ This configuration using a BPM provides ample possibilities in the cell design, since an electrolyte with a different pH can be used at either side; *e.g.* a near-neutral pH environment at the cathode against a high pH at the anode.^14^ The reverse bias mode is traditionally used in bipolar membrane electrodialysis (BPMED)^15,16^ and commonly used for energy applications (including CO_2_ reduction and water splitting)^14,17^ and resource recovery.^18^ Li *et al.* demonstrated better stability when sandwiching a BPM between gas diffusion electrodes (GDEs), compared to monopolar membranes, at various current densities.^8^ Salvatore *et al.* reached a faradaic efficiency (FE) of 50% at 200 mA cm^−2^ with a liquid support layer of NaHCO_3_ between the BPM and GDE.^9^ However, in neutral pH, the overpotential of the WDR increases significantly. On the other hand, when the cathodic catalyst layer is attached to the CEL of the BPM directly in an MEA configuration, an acidic environment surrounds the catalyst, possibly favouring the unwanted hydrogen evolution reaction.

The second operating mode is forward bias with the AEL facing the cathode, where ions are transported towards the interface where recombination or precipitation can occur.^19^ In the forward bias mode, water is being formed at the interface layer, which can cause blistering.^20,21^ This configuration was proposed for CO_2_ reduction^10^ to leverage the recent achievements in AEM-based MEAs.^22,23^ Although ions may accumulate at the BPM interface layer,^24,25^ the cathode–AEL environment could be combined with a low membrane overpotential in the forward bias mode. Pătru *et al.* demonstrated a forward bias system (vapour–vapour) reaching a FE towards CO of 13% at 50 mA cm^−2^ while inhibiting CO_2_ crossover to the anode.^10^ This unwanted crossover of CO_2_ (and its negatively charged dissolved species, CO_3_^2−^ and HCO_3_^−^, or carbonic acid, H_2_CO_3_, which are all grouped under the term dissolved inorganic carbon, DIC) compromises the efficiency of CO_2_ electrolyzers and is a well-known problem, especially in AEM-based MEA configurations.^26^

In general, both biases of a BPM showed stability in the order of a few hours. For developing BPM-based CO_2_ electrolyzers, long term stability and therefore low ion and product crossover through the membrane are essential. In order to achieve long term stability for both BPM orientations in an MEA-based CO_2_ electrolyzer, knowledge of the ion transport is needed, which is currently lacking in a vapour–liquid environment. In particular, little is published on the transport mechanisms in the forward bias mode. In this study, we reveal the ion transport mechanisms and practical feasibility of a BPM in reverse bias and forward bias embedded in an MEA for CO_2_ electrolysis.

## Results and discussion

To study the different transport mechanisms of ions and carbonic species within a BPM-based MEA (BPMEA), we examine two cases for CO_2_ electrolysis, one case using a BPM in reverse bias and another case in forward bias. In order to preferentially make gaseous products, Ag was used as a catalyst which was directly sputtered onto the membrane. Ag has shown the ability to reduce CO_2_ to CO and H_2_ with different product ratios depending on the applied potential, electrolyte, and pH.^1^ The use of a catalytic layer deposited directly on the membrane, in the absence of a carbon based porous diffusion layer, allows the ability to observe the possible salt formation (*i.e.*, occurring due to transportation of the electrolyte ions through the BPM towards the catalyst). To achieve a direct deposition on the membrane *via* sputtering in a vacuum, and to avoid structural changes in the membrane moiety, which would occur if absorbed water vaporizes, a heterogeneous Ralex® bipolar membrane was used in its dry state as the catalyst support. During the sputtering process, the BPM was de-aerated, creating micro-cracks which facilitate the crossover of CO_2_ (see later). A description of the fabrication process can be found in the ESI.†

**Fig. 2:**
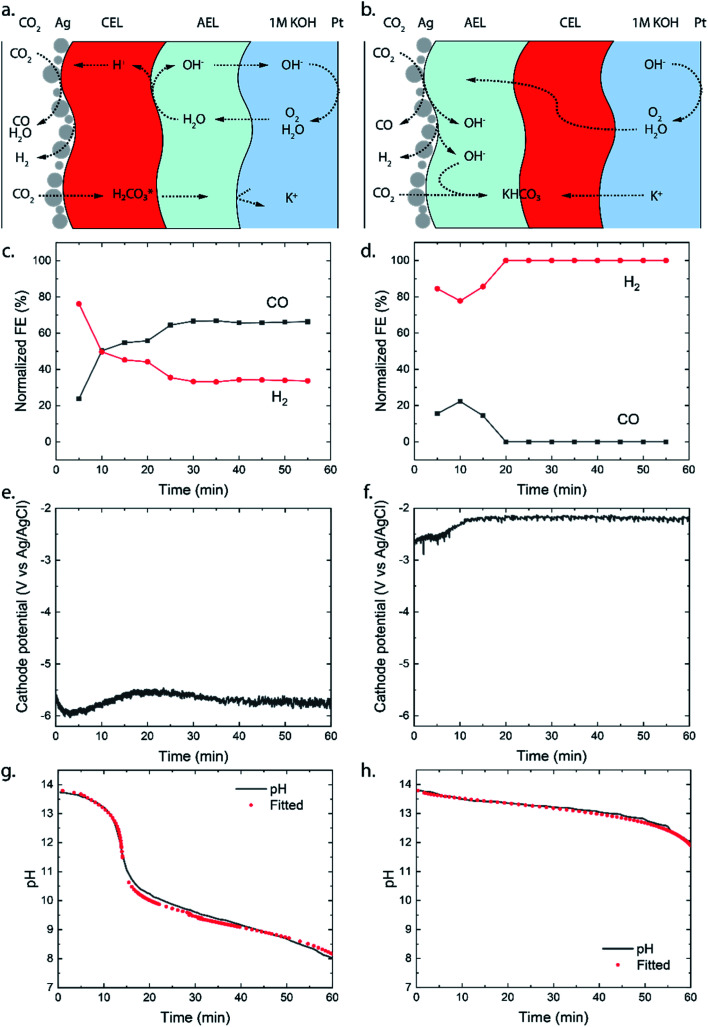
Schematic illustration of transport of water, CO_2_ and ionic species in (a) the reverse and (b) the forward bias mode. The catalyst at the cathode is Ag, and at the anode it is Pt. The normalized faradaic efficiencies are shown for (c) the reverse and (d) the forward bias mode. Normalization to the total evolved product is needed to compensate for the varying gas flow rate. Graphs (e and f) show the cathodic potentials at a current density of 25 mA cm^−2^ (1.56 cm^2^ surface area, 0.036 cm^3^ s^−1^ flow rate with 4 mm electrolyte spacing and the reference electrode (3.4 M KCl, 240 mV *vs.* SHE) in the anolyte) for each mode and the graphs (g and h) show measured pH in the anolyte with a fitted pH based on the molar flux of dissolved inorganic carbon species. The anolyte is initially 1 M KOH.

A diagram of the BPMEA in reverse bias mode is illustrated in Fig. 2a. Upon applying a current, water is dissociated into protons (H^+^) and hydroxide ions (OH^−^) in between the CEL and AEL. OH^−^ will then transport through the AEL into the electrolyte to replenish the consumed hydroxide ions at the Pt anode (resulting in the oxygen evolution reaction). For the forward bias mode (Fig. 2b), the Ag catalyst was deposited on the AEL, where anions (*e.g.*, (bi)carbonate) migrate towards the interface with the CEL. Similarly, cations migrate in the CEL in the opposite direction. Hence, the hypothesis for the forward bias is that no ion depletion occurs at the internal BPM interface and therefore no net WDR is expected, but salt accumulation occurs instead. The implications of each of these two different charge transport mechanisms will be discussed in the following sections.

The selectivity of the cathodic reaction in our MEA vapour–liquid configuration depends on the orientation of the bipolar membrane. The reverse bias demonstrates a stable CO production (60% FE) for one hour of operation as shown in Fig. 2c, after an initial stabilisation period where more H_2_ was produced. With an opposite membrane orientation (forward bias mode), a significantly lower selectivity was obtained: initially some CO was produced (maximum FE of 20%), whereas CO could no longer be detected after 20 minutes and only H_2_ was observed (Fig. 2d). As we will explain further on, the ion transport mechanism changes around 20 minutes of operation, and this also influences the selectivity at the cathode. The experiments were performed in triplicate to observe possible sample-to-sample variation, and similar maximum FEs were obtained in each case with the exception that one sample in the forward bias mode, with little CO_2_ crossover, showed a continuous CO production of 10% (see Fig. SI2†).

**Fig. 3:**
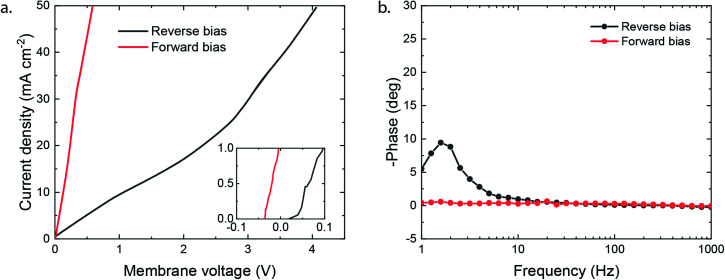
(a) Linear sweep potentiometry at 0.5 mA s^−1^ in 1 M NaCl (corrected for electrolyte losses) with positive membrane potential indicating reverse bias and negative forward bias; values at 25 mA cm^−2^ are 2.74 V (reverse) and 0.41 V (forward). (b) Galvanostatic impedance at 25 mA cm^−2^ with 6.4 mA cm^−2^ amplitude.

The potential required to reach the applied 25 mA cm^−2^ strongly differs depending on the membrane orientation. For the reverse bias mode, with the cathodic potential shown in Fig. 2e, a highly negative potential (up to −5.5 V *vs.* Ag/AgCl in the anolyte) was needed to perform the cathodic reactions, while for the forward bias mode a cathodic potential of around −2.0 V *vs.* Ag/AgCl was required to achieve the same current density (Fig. 2f). The major difference can be explained from the processes in the interfacial layer between the CEL and AEL. The main energy loss in the reverse bias mode appears to be associated with the WDR, shown by the membrane voltage difference of 2.3 V at 25 mA cm^−2^, when reverse bias is applied compared to forward bias (see Fig. 3a). In addition, the impedance of the BPM measured in each orientation supports the hypothesis that the WDR is (almost completely) absent in forward bias: the peak related to the WDR disappears in comparison to reverse bias (see Fig. 3b).^13^ The voltage required to drive the WDR indicates that the catalyst at the internal interface is kinetically slow. Because our membrane was chosen to allow synthesis in a dry state, this particular commercial BPM is not optimized for CO_2_ reduction in this configuration. Recent literature has shown *via* experimental work^27–29^ and simulations^30,31^ that the catalyst overpotential can be easily reduced by two orders of magnitude at these current densities compared to the one that was used for these studies. Hence, the cathode potentials in Fig. 2e and f are expected much closer to their thermodynamic equivalent in an optimized BPMEA system.

As no net WDR occurs in the forward bias mode and ion transport is directed towards the internal interface, ions can neutralize in the BPM. The type of neutralization differs depending on the type of ion, *e.g.* protons and hydroxide ions will perform water association and allow harvesting a significant membrane voltage due to the high gradients in the H^+^/OH^−^ concentration over the CEL/AEL interface, leveraged in BPM-based batteries.^16,32^ However, only limited protons are present in the CEL in the forward bias mode, and K^+^ is present in abundance instead (as the anolyte is chosen to be KOH). In the case of such alkaline CO_2_ electrolysis, K^+^ will neutralize the anions (*i.e.*, the carbonate ions entering the AEL from the gas side) at the internal interface, which will in theory generate a relatively small potential drop of 59.1 mV for every order of magnitude difference in the concentration across the interface. Based on the work of Strathmann and co-workers, the concentration in the membrane layers was found to be a function of the charge density of the BPM (which is experimentally determined to be 3.2 M, see the ESI†) and the concentration of ions in the electrolyte.^33^ The potential drop can then be calculated based on the concentration of K^+^ in the CEL (3.5 M) and in the AEL (0.3 M), resulting in a potential drop of 64 mV. This salt neutralization would imply a reduced cell voltage, also indicated by the negative membrane voltage at low current density in forward bias (Fig. 3a).

Although carbonate species may be the dominant charge carriers through the BPM layers, the crossover of CO_2_ seems to be lower in the forward bias than in the reverse bias, derived from the pH of the anolyte, as illustrated in Fig. 2g and h. Initially, the OH^−^ is consumed to turn CO_2_ into CO_3_^2−^ (*via* HCO_3_^−^) as shown in eqn (1) and (2).

1 CO_2_ + OH^−^ → HCO_3_^−^, p*K*_a_ = 6.3

2 HCO_3_^−^ + OH^−^ → CO_3_^2−^ + H_2_O, p*K*_a_ = 10.3

Once the pH of the anolyte decreases below the p*K*_a_ shown in eqn (2), after approximately 15 minutes, only the reaction shown in eqn (1) will proceed in the right hand direction, while the reaction shown in eqn (2) is reversed since the equilibrium conditions change.

Eqn (1) and (2) allow estimation of the CO_2_ crossover from the experimentally observed change in pH for each mode. Chemical equilibrium software, Visual MINTEQ, was used to fit the molar flux of carbonic species through the entire BPM. A constant flux did not give a good fit with the experimental measurements, which seems reasonable since the conditions in the cell change over time as the carbonic species get absorbed by the KOH anolyte. The gradual build-up of DIC in the anolyte leads to a lower concentration gradient over the BPM, which lowers the DIC flux over time (see Fig. SI3†).

For the reverse bias mode, a rapid pH change is observed during the experiment as a pH of 8.0 is reached after 60 minutes. The forward bias mode reaches a pH of 12.0 after only 60 minutes, indicating that the OH^−^ is consumed at a rate approximately 5× slower than that in the reverse bias. It is important to note that in the forward bias mode no net WDR occurs and therefore no replenishment of consumed OH^−^ ions at the anode takes place. The consumption of OH^−^ at the anode accounts for 15% of the OH^−^ loss.

To explain the stark difference in CO_2_ crossover depending on the BPM orientation, we need to realize *via* which species the CO_2_ dissolves and migrates through the BPM membrane layers. For the reverse bias mode, where the CEL is adjacent to the catalyst, the majority of the mobile species in the CEL have a positive charge. There is a flux of protons coming from the internal membrane interface, resulting in a pH below 7 in the CEL. Therefore, the CO_2_ (g) will dissolve and transport as carbonic acid (H_2_CO_3_ (aq) or CO_2_ (aq)) through the CEL, as illustrated in Fig. 2a. Once the carbonic species cross the internal membrane interface due to diffusion, the environment becomes more alkaline due to the high concentration of hydroxide ions either from the anolyte or from the WDR. Here, the carbonic acid will be converted into (bi)carbonate species. These (bi)carbonate species will move further into the anolyte and consume OH^−^ ions.

The forward bias mode causes the pH to be higher than 7 at the catalyst–membrane interface, since the AEL prevents high concentrations of protons and the CO_2_RR produces a significant amount of OH^−^. The carbonic species will enter the AEL layer, depending on the actual pH, as carbonic acid, bicarbonate and/or carbonate. If the pH is higher than 8.5, then the formation of bicarbonate is dominant compared to that of the carbonic acid as the CO_2_ (g) will react directly with OH^−^.^34^ These carbonic species will then move towards the internal membrane interface. At the internal interface, the bicarbonate ions will be rejected by the negative fixed charges of the CEL. Unlike in the case of the reverse bias mode, where the CEL is being fed with protons (produced from the WDR at the interface layer), no source for H^+^ is present in the forward bias. Therefore, we hypothesize that the (bi)carbonate species cannot easily cross the CEL in an electrolysis cell with an alkaline anolyte. Carbonic acid will not be formed due to the high pH in the AEL, and the (bi)carbonate species are rejected by the positive fixed charges in the AEL. This behaviour explains the mild pH change in forward bias shown in Fig. 2g.

**Fig. 4:**
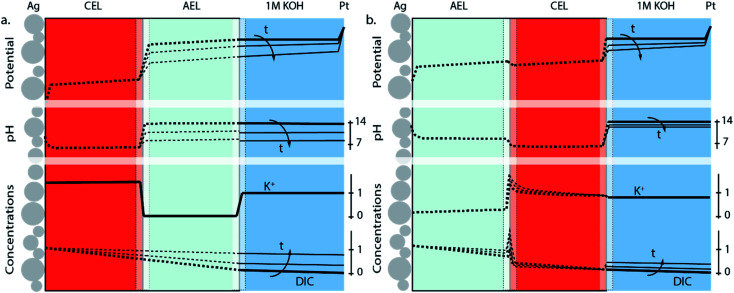
Potential gradient, pH and concentration profiles of K^+^ and dissolved inorganic carbon (DIC) species at a fixed current density with CO_2_ leakage through the BPM for (a) the reverse bias and (b) forward bias orientation. For graphical clarity, the membrane–membrane and the membrane–electrolyte interface are shown at an increased scale. Solid lines are the experimentally obtained values, while the dotted ones are illustrative approximations.

The differences in concentration profiles between reverse and forward bias are summarized in Fig. 4, along with the potential and pH of the electrolyte layer, AEL and CEL. For the reverse bias case, a jump in pH and potential (due to the WDR) occurs at the membrane–membrane interface (Fig. 4a) when a current is applied, which is qualitatively based on recent simulations.^31^ As the pH in the AEL and anolyte decreases, the required potential across the membrane–membrane interface reduces, but is compensated at the anode. Over time, the flux will decrease as the DIC concentration gradient over the CEL decreases. The distribution of K^+^ remains approximately constant over time, and the concentration at the CEL is similar to the ion exchange capacity. For the forward bias case (Fig. 4b), there is no increase in potential at the AEL–CEL interface when going from the AEL to CEL; instead, a small decrease in potential exists due to the recombination of salt and potentially water in the membrane. This is also reflected in the concentration polarization of K^+^ in the CEL near the membrane–membrane interface, and the concentration polarization of (bi)carbonate in the AEL (Fig. 4b). Due to the water association and salt accumulation in forward bias, a substantial pH difference between the AEL and CEL is absent, which limits the concentration of DIC in the CEL. Hence, the change of the pH, DIC and potential of the anolyte is slower over time in the case of forward bias (Fig. 4b) than that in reverse bias (Fig. 4a).

**Fig. 5:**
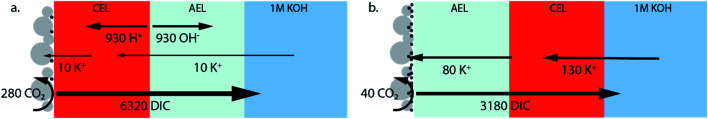
Mass balance of the WDR products, K^+^ ions, CO_2_ conversion and DIC crossover for (a) the reverse bias and (b) forward bias orientation. The values are in μmol cm^−2^ which is the total number exchanged after the duration of the experiment (1 h). Calculations of the ionic mol balances are described in the ESI.†

Due to the high concentrations of K^+^ and (bi)carbonate at the AEL–CEL interface in the forward bias mode, ion interaction could occur, possibly leading to salt formation at the membrane interface. This hypothesis is formed based on fuel cells with a BPM, where transport of H^+^ and OH^−^ ions to the interface is reported and thus water recombination occurs at the interface.^20^ However, in the case of CO_2_ electrolysis, few protons are available and water recombination will not occur; therefore, it is suggested that other reactions, such as the formation of potassium salts, occur. Although this AEL–CEL voltage brings a slight negative contribution to the total cell voltage, it will compromise the chemical potential difference over time, as the electrolyte loses its ionic strength. The increase of K^+^ in the membrane layers is confirmed for the forward bias mode *via* inductively coupled plasma-optical emission spectroscopy (ICP-OES) measurements before and after the experiment, as shown in the mass balance in Fig. 5. In the forward bias mode, the K^+^ ions migrate at a rate of 130 μmol cm^−2^ per hour, of which 80 μmol cm^−2^ is transported to the catalyst surface. The remaining 50 μmol cm^−2^ after one hour implies that the total K^+^ concentration is doubled (to twice the ion exchange capacity), while the amount of mobile charges in the membrane remains constant in the reverse bias mode.

In this study we also focus on the crossover of K^+^ from the anolyte to the catalyst at the cathode where it can form salts, which is a common issue in MEAs for CO_2_ electrolysis.^35^Fig. 5a and b show the mass balance for each configuration of the BPM. The crossover in the reverse bias mode does follow the same crossover rate as when the bipolar membrane is placed in a liquid–liquid interface (at 25 mA cm^−2^, 7 μmol h^−1^ cm^−2^).^11^ As mentioned earlier, the flux of K^+^ through the CEL is significantly higher in the forward bias mode due to the neutralization of the carbonic species. In addition, the K^+^ deposits almost 8 times faster at the Ag catalyst layer in the forward bias than in the reverse bias, while the K^+^ needs to pass the exact same membrane layers (only in a different order). The strongly enhanced K^+^ crossover could be due to the higher concentration of K^+^ in the CEL near the CEL–AEL interface, leading to a higher concentration gradient over the AEL. Another hypothesis is the diffusion of dissolved salt (KHCO_3_ or K_2_CO_3_), formed at the internal interface, towards the cathode.

A parameter that would influence the ion crossover – in addition to the orientation, current density and electrolyte composition – is the thickness of the membrane layers. As we prepared our membrane electrode assemblies based on a commercial bipolar membrane (heterogeneous Ralex® bipolar membrane), it was not possible to change the thickness. However, based on the literature we can already estimate what the consequences would be if the thickness is altered. Recent simulations by Bui *et al.* (2020) and experimental work by Mayerhöfer *et al.* (2020) showed that the thickness of the anion exchange layer (AEL) is of main influence on the ion crossover.^31,36^ Although the influence of membrane thickness on the crossover of (uncharged) CO_2_ is not studied previously, we can hypothesize that in reverse bias the CO_2_ crossover is insensitive to the AEL thickness as the CEL determines the speciation of CO_2_ and forms the main barrier for carbon transport. However, the K^+^ transport would increase if the AEL thickness were reduced.^31^ Similarly, for the forward bias case, we would expect that both carbon transport and more K^+^ crossover would increase as the AEL thickness reduces. At the same time, a thinner membrane could strongly enhance the conductance of the membrane, lowering the energy losses. To break this trade-off, a more active interlayer catalyst (enhancing the WDR) is required. Given that the much thinner FumaSep® bipolar membranes (<200 μm) feature much lower resistance and similar relative cation crossover^11^ compared to the Ralex® membrane, thinner membranes with a more active WDR catalyst seem a realistic approach to improve the system. The effort to test this hypothesis, *via* reproducing BPMs at different thicknesses, is out of the scope of the study.

Fig. 5 also reveals that the earlier discussed CO_2_ crossover is at least one order of magnitude larger than the flux of potassium. For the reverse bias mode, the amount of CO_2_ crossing over is 36% of the CO_2_ feed (10 mL min^−1^), while only 1.6% is consumed at the cathode. For the forward bias mode, these values are 18% and 0.3% at maximum FE, respectively. A physical description for the high crossover in both cases is the deposition method of the Ag, *via* vacuum sputtering, which de-aerates the BPM, creating micro-cracks that facilitate the crossover of the CO_2_. These micro-cracks did not penetrate both layers, but most likely reduced the physical barrier for CO_2_ of one membrane layer, increasing the diffusion coefficient of the carbonic species through these layers.

The combination of observed micro-cracks and high cell voltages in the reverse bias mode (Fig. 2e), low faradaic efficiency in the forward bias mode (Fig. 2d), and the literature results from Pătru *et al.*^10^ could be associated with water management in the membrane layers. The water dissociation in the reverse bias mode may dehydrate the membrane layer, which provides a suitable faradaic efficiency (Fig. 2c), but increases the membrane voltage (Fig. 2e) and CO_2_ crossover due to micro-cracks. At the same time, the forward bias mode, without water dissociation or even water recombination at the membrane interface, may suffer from a too high water content that compromises the faradaic efficiency. The latter effect has been observed as well by Pătru *et al.*^10^ To test this hypothesis, a BPMEA was pre-treated by soaking it in demi water for 24 hours, which resulted in a 100% FE for H_2_ in the reverse bias mode (see Fig. SI5†).

The high overpotentials and CO_2_ crossover, and water management of membrane layers, show that a different BPM is required to optimize the absolute performance for CO_2_R in this catalyst integrated MEA. In this work, the architecture was chosen to understand the ion transport mechanisms with the different orientations of the BPM, allowing the ability to assess the ion accumulation at the surface(s). However, a BPM for an MEA in practical CO_2_ electrolysis should possess a higher catalytic energy efficiency and ionic conductance. Also for the deposition method, spray deposition could be used as a less destructive technique than the vacuum sputtering used in this study. The spray deposition often requires a conductive support, such as a gas diffusion electrode. Considering such a practical architecture, the salt formation that was observed in the forward bias mode can facilitate flooding when used in combination with a gas diffusion electrode, impacting the performance of the system.^35^ In general, forward bias operation has intrinsic instability issues in terms of salt accumulation, which limits the operational lifetime of the BPM from hours to days.^10^ Reverse bias operation suffers from significant CO_2_ crossover and requires a very high membrane voltage (see Fig. SI4†), but these aspects can be tuned by engineering more robust, thin membrane layers and better WDR catalysts for optimized CO_2_ electrolyzers. Furthermore, despite the acidic conditions of the cathode in reverse bias, the faradaic efficiency for CO is still considerably higher than that in the forward bias mode, even when the catalyst is sputtered directly on the (acidic) CEL.

## Conclusions

We have studied the transport mechanisms of ions and (both charged and neutral) carbonic species as a function of the orientation of a bipolar membrane within a MEA performing electrochemical CO_2_ reduction. A BPM-based MEA with the reverse bias orientation was compared to the opposite orientation, forward bias. Both orientations had a vapour–liquid cell configuration where a CO_2_R catalyst (Ag) was directly deposited on the membrane interface. For the reverse bias mode, where the cation exchange layer of the BPM is in contact with the catalyst, the dominant ion transport mechanism is water dissociation that occurs at the internal membrane interface. At the same time, CO_2_ crosses over through the BPM, starting by entering the CEL as carbonic acid (due to the low pH in the CEL) and being transported through the AEL towards the Pt anode as (bi)carbonate. The molar flux of CO_2_ that crosses over through the BPM decreases over time due to the reduction in the dissolved inorganic carbon (DIC) concentration gradient.

For the case where the BPM is placed in the forward bias orientation (*i.e.*, the CEL facing the anode), no water dissociation reaction occurs, saving 3 V in the cathodic potential at 25 mA cm^−2^ compared to the reverse bias mode. The molar flux of carbonic species is half of that in the reverse bias mode and has a similar decreasing trend over time. After 10 minutes of operation, a reduction in the absolute cathodic potential is observed which coincided with a decreased selectivity towards CO evolution. We hypothesize that these observations indicate salt accumulation and higher water content due to the absence of water dissociation in the internal membrane interface, which are intrinsic to the forward bias operation. Because of the salt build-up and high concentration of K^+^ and (bi)carbonates at the CEL–AEL interface, more K^+^ can cross over through the AEL, depositing on the catalyst surface. Our study shows that there are performance trade-offs for each BPM orientation with regard to potential, selectivity, and stability: the forward bias lowers the overall cell potential by reducing the chemical potential, while the reverse bias gives a stable product formation of CO_2_ conversion products.

## Conflicts of interest

There are no conflicts to declare.

## Supplementary Material

TA-009-D0TA12398F-s001
