# Supplementary material for: Orientation of a bipolar membrane determines the dominant ion and carbonic species transport in membrane electrode assemblies for CO2 reduction
Source: J Mater Chem A Mater. 2021 Mar 11;9(18):11179–86. doi: 10.1039/d0ta12398f (PMC8112234; doi:10.1039/d0ta12398f)
Supplement: TA-009-D0TA12398F-s001 [file TA-009-D0TA12398F-s001.pdf]

Electronic Supplementary Material (ESI) for Journal of Materials Chemistry A.
This journal is © The Royal Society of Chemistry 2021

Supporting information

for

Orientation of bipolar membrane determines dominant ion and carbonic species transport in membrane electrode assemblies for CO_2 reduction

Marijn A. Blommaert[1], Rose Sharifian[1,2], Namrata Shah[1], Nathan Nesbitt[1], Wilson A. Smith[1] , David A. Vermaas[1] *

[1]Department of Chemical Engineering, Delft University of Technology, 2629 HZ Delft, The Netherlands

[2]Wetsus, European Centre of Excellence for Sustainable Water Technology, 8911 MA Leeuwarden, The Netherlands

Fabrication of BPMEA

A heterogeneous dry bipolar membrane (Ralex® BM, MemBrain s.r.o.), with a thickness of 460 µm, was used to fabricate the bipolar membrane electrode assembly (BPMEA). We opted for the Ralex membrane as it is produced dry, allowing to be vacuum sputtered on the surface. On the membrane, a 100 nm layer of Ag was deposited (50 Watt DC power, 20 sccm argon and 3 microbar pressure; sputter deposition time was 306 seconds). The same method was used for the reverse bias case and the forward bias case. The rough structure of the membrane layers leads to holes in the Ag layer (see SEM figure, Fig SI1). Once the membrane starts to swell and is electrochemically active, the Ag layer is no longer conformal and has small ruptures. These gaps in the Ag layer are actually required to facilitate the transport of ions and CO_2 to the active sites.

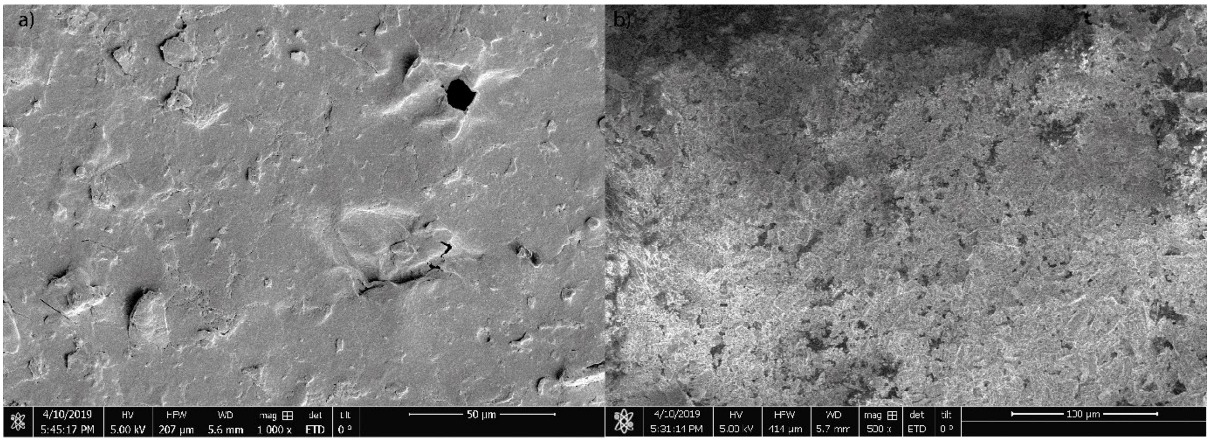

Figure SI1: SEM-image of the sputtered Ag-layer on the BPM in (a) dry state and (b) after CO_2 reduction in reverse bias case.

Electrochemical experiments

The experiments were performed in a flow cell (MicroFlowCell, purchased from Electrocell, Denmark). The catalyst on the BPMEA was fixed at the flow cell with copper tape, which also served as current

collector. The catalyst surface area was 1.56 cm^2, the catalyst at the anode (Ni) had a similar surface area. Between the BPMEA and the anode, an anolyte was flushed around (0.035 $cm^3\ s^{-1}$). A micro reference electrode of type LF-1-100 (manufactured by Innovative Instruments Inc. with a polyetheretherketone (PEEK) body and a diameter of 1 mm) was inserted in the anolyte to measure the cathode potential. Just before the experiment was started, H_2O was flown in the anolyte, in order to wet the membrane. This wetting allowed the membrane to swell, reducing the resistance of the BPMEA.

Faradaic efficiency of forward bias case with stable CO production

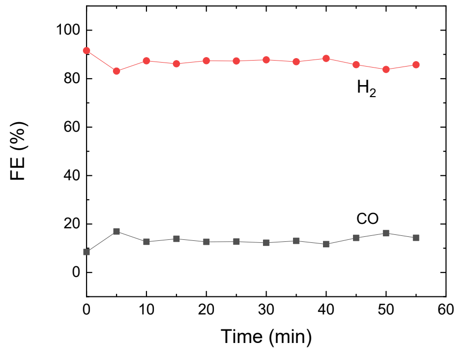

Figure SI2: Sample of forward bias mode with continued CO production.

Calculation charge density and membrane concentration

The charge density of the Ralex membrane was determined via the procedure described by Galama *et al.*[1] Only the cations (Na, resp.) were measured via ICP-OES. The amount of Na present in the CEL was 84.06 µmol. With a water uptake of 0.84 g H_2O/g for the entire membrane (assuming that for the CEL this is half of the water uptake, 0.42 g H_2O/g), and an ion exchange capacity of 0.0013 mol/g, this leads to a charge density of 3.18 mol/kg H_2O.

The concentration in the membrane layers during operation were determined via Strathmann *et al.*[2] With the use of following formula, one can calculate (for the reverse bias mode) the concentrations in the CEL, knowing that $C_{K^+,bulk} = 1$:

$$C_{K^+,CEL} = \frac{X + \sqrt{X^2 + 4C_{K^+,bulk}}}{2} = 3.48$$

And in the AEL:

$$C_{K^+,AEL} = \frac{X - \sqrt{X^2 + 4C_{K^+,bulk}}}{2} = 0.28$$

Molar flux calculation

Molar flux of DIC is calculated based on the consumption of the hydroxide ions and therefore its concentration.

$$[OH^-]_{consumed} = 10^{-(pK_W - pH_1)} - 10^{-(pK_W - pH_2)}$$

Where pH_1 and pH_2 are two values between a certain time, t, in which the molar flux should be calculated with z_i the valence of the DIC species dominating at that time:

$$J_{DIC} = {[OH^-]_{consumed}}/{z_i \cdot t} \quad [{M}/{min}]$$

Due to the high salinity of the 1M KOH, the pK_W is not exactly 14, but around 13.65.

Dissolved inorganic carbon

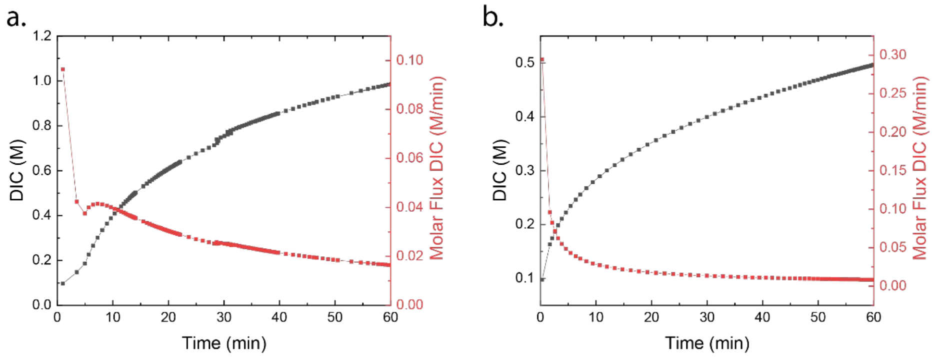

Figure SI3: DIC and molar flux of DIC in function of time of (a) reverse bias mode and (b) forward bias mode.

Calculation mass balance (figure 5 main article)

Based on the applied charge throughout the operation (t=1 hr, i=25 mA cm^{-2}, A=1.56 cm^2):

$$Q = i * t * A = 140.4\ C$$

One can calculate the amount of protons consumed at the cathode:

$$\#\ protons = \frac{Q}{96485} = 0.00145\ mol$$

Divided by time and surface area one finds the flux, which should be the same at the cathode as at the internal BPM interface:

$$J_{protons} = \frac{0.00145\ mol}{1\ hr * 1.56\ cm^2} = 932.8\ \mu mol\ hr^{-1}\ cm^{-2}$$

The crossover of K^+ is determined via ICP-OES in mol and via a similar method converted to a flux.

To determine the amount of CO_2 converted by the cathode, the amount of charge is multiplied by the faradaic efficiency of each case and then converted into a flux.

For the total amount of CO_2 that has crossed over through the membrane, the DIC concentration at the experiment (see Fig SI3) was converted from mol l^{-1} to a flux, knowing that the anolyte concentration was 10 ml.

Potential distribution of both orientations

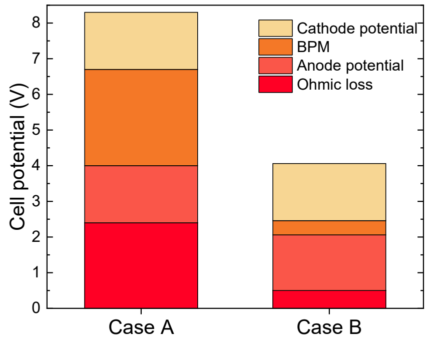

Figure SI4: Potential breakdown for the setup with reverse bias orientation (case A) and forward bias (case B). The experiments in triplicate to cancel out sample-to-sample variation; cell voltage of reverse bias has a standard deviation of 0.43 V and forward bias of 0.24 V.

Wetting of bipolar membrane

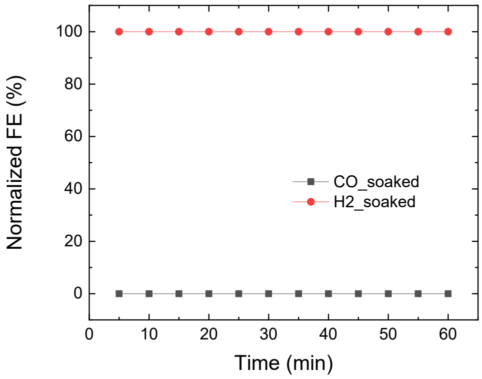

Figure SI5: Effect of preconditioning the BPMEA on the normalized Faradaic efficiencies by soaking the membrane with catalyst for 24 hours in demi water, at 25 mA cm^{-2} in reverse bias mode.
